# ECG-Mamba: Cardiac Abnormality Classification With Non-Uniform-Mix Augmentation on 12-Lead ECGs

**DOI:** 10.1109/JTEHM.2025.3613609

**Published:** 2025-09-23

**Authors:** Huawei Jiang, Husna Mutahira, Shibo Wei, Mannan Saeed Muhammad

**Affiliations:** Department of Computer Science and EngineeringSungkyunkwan University 35017 Suwon 16419 South Korea; Department of Computer Science and EngineeringSogang University 35014 Seoul 04107 South Korea; Department of Biomedical Science and EngineeringGwangju Institute of Science and Technology 65419 Gwangju 61005 South Korea; School of Electronic Engineering and Computer ScienceQueen Mary University of London 4617 E1 4NS London U.K.

**Keywords:** Heart abnormality detection, ECG-Mamba, non-uniform operations, non-uniform-mix

## Abstract

Objective: The detection of heart abnormalities using electrocardiograms (ECG) is a critical task in medical diagnostics. A lot of literature has utilized ResNet and Transformer architectures to detect heart disease based on ECG signals. Recently, a new class of algorithms has emerged, challenging these established methods. A selective state space model (SSM) called Mamba has exhibited promising potential as an alternative to Transformers due to its efficient handling of longer sequences. In this context, we propose a Mamba-based model for detecting heart abnormalities, named ECG-Mamba. Recognizing that common data augmentation methods such as MixUp and CutMix do not perform well with Mamba on ECG data, we introduce a data augmentation technique called non-uniform-mix to enhance the model’s performance.Methods and procedures: ECG-Mamba is based on Vision Mamba (Vim), a variant of Mamba that utilizes a bidirectional SSM, enhancing its capability to process ECG data effectively. To address the sensitivity of the Mamba model to noise and the lack of suitable data augmentation techniques, we propose a data augmentation algorithm that conservatively introduces data augmentation by performing non-uniform operations on the dataset across different epochs. Specifically, we apply MixUp to a portion of the dataset in different epochs.Results: Experimental results indicate that ECG-Mamba outperforms the best algorithms in the PhysioNet/Computing in Cardiology (CinC) Challenges of 2020 and 2021 based on the AUPRC and AUROC, specifically with ECG-Mamba achieving an AUPRC score 16.6% higher than the best algorithm in the PhysioNet/CinC Challenge 2021 on 12-lead ECGs, reaching 0.61. Moreover, with the proposed data augmentation method Non-Uniform-Mix, ECG-Mamba’s AUPRC reached 0.6271, representing a 2.8% improvement.Conclusion: The ECG-Mamba model, based on the SSM, demonstrates potential in detecting cardiac abnormalities from ECG data. Although the model surpasses existing algorithms, it exhibits sensitivity to noise, requiring careful data augmentation. The proposed conservative data augmentation technique addresses this challenge and improves the model’s performance, suggesting a promising direction for future research in ECG analysis using SSMs. The implementation is publicly available at https://huggingface.co/poult/ECGMambaVersionOfJTEHM2020-2021_final.Clinical and Translational Impact Statement: ECG-Mamba enhances heart abnormality detection, enabling early diagnosis and personalised treatment in resource-limited and telemedicine settings. Using real-world data from the PhysioNet/CinC Challenges 2020 and 2021, it accurately models multiple concurrent cardiac conditions, reflecting complex clinical scenarios. Its conservative Non-Uniform-Mix augmentation mitigates noise sensitivity, improving accuracy and reliability for seamless integration into clinical workflows, thus supporting evidence-based practice and addressing healthcare disparities.

## I. Introduction

Electrocardiograms (ECG) signals are pivotal in diagnosing heart abnormalities. They offer a non-invasive and efficient method for monitoring cardiac health. With cardiovascular diseases remaining a leading cause of mortality worldwide and the growing demand for rapid and accurate diagnostic tools [1], improving ECG analysis capabilities has become crucial for modern healthcare systems. Furthermore, the increase in wearable ECG devices and remote monitoring systems has generated vast amounts of cardiac data requiring efficient processing and analysis [2].

With the increasing availability of large ECG datasets, neural network techniques have become essential for improving the accuracy and efficiency of heart abnormality detection [3], [4], [5], [6]. Automated ECG interpretation not only alleviates the burden on healthcare professionals but also improves diagnostic accuracy and reduces variability, particularly in resource-limited settings where access to expert cardiologists is limited.

Traditional neural network models, including Convolutional Neural Networks (CNNs) and Transformers, have demonstrated considerable success in this domain [7], [8]. However, these models often face challenges when dealing with the long sequence data inherent in ECG signals, [9]. Recently, selective state space models (SSMs), particularly the Mamba [10], have emerged as promising alternatives due to their superior ability to manage longer sequences effectively. Mamba is mainly used for tasks such as language modelling and time-series prediction. Vision Mamba (Vim) [11], an adaptation of the Mamba architecture for visual data processing, integrates SSMs with visual sequence encoding.

In this paper, a Mamba-based model, termed ECG-Mamba, is introduced to process 12-lead ECG signals. ECG-Mamba builds upon Vim, a bidirectional SSM, and is specifically tailored to detect heart abnormalities from ECG data. Experimental results indicate that ECG-Mamba outperforms leading algorithms ISIBrno [12], Prna [13], and ISIBrno without attention [14], from the PhysioNet/Computing in Cardiology (CinC) Challenges of 2020 and 2021 in terms of AUPRC and AUROC on public datasets, highlighting its potential as a robust tool in medical diagnostics.

The Vim architecture demonstrated promising results but exhibited sensitivity to noise, making it difficult to apply conventional data augmentation techniques such as MixUp [15] and CutMix [16] without compromising performance. Additionally, traditional data augmentation methods like random cropping, merging, horizontal flipping, and vertical flipping can be detrimental to ECG classification performance, as they alter the signal in ways that are not physiologically meaningful, effectively adding noise [17]. For instance, random cropping and merging can transform a normal sinus rhythm into an arrhythmic pattern, introducing noise rather than meaningful data [18]. Therefore, it is critical to design augmentation methods that attenuate the influence of noise on the model while simultaneously improving performance on unseen data, particularly for the Mamba-based architecture proposed in this study.

To address this, the proposed data augmentation strategy applies augmentations conservatively by performing non-uniform operations on the dataset across different epochs. Specifically, MixUp is selectively applied to different parts of the dataset in each epoch, minimizing noise while preserving data integrity. This tailored approach has the potential to improve the performance of Mamba structures by introducing noise in a controlled manner.

The contributions of this paper are as follows:

• The paper introduces ECG-Mamba, an adaptation of the Vim architecture for processing 12-lead ECG signals, providing a benchmark for effective heart abnormality detection.
• It demonstrates that ECG-Mamba outperforms leading algorithms from the PhysioNet/CinC Challenges of 2020 and 2021 in terms of AUPRC and AUROC on public datasets, highlighting its potential in medical diagnostics.
• A tailored data augmentation strategy is developed to address the Mamba architecture’s sensitivity to noise, thereby enhancing ECG-Mamba’s robustness and performance.

## II. Related Work

### A. ECG Classification Based on Deep Learning

Recent advancements in ECG classification have shown promising results using deep learning techniques. The method in [12], which secured first place in the PhysioNet/CinC Challenge 2020, utilizes transformer models to capture long-range dependencies in ECG signals. The approach in [13], the winner of the PhysioNet/CinC Challenge 2021, employs residual networks and attention mechanisms for enhanced feature extraction. Subsequent work in [14] achieved a 2% improvement in AUPRC on the 12-lead dataset compared to [13] by removing the attention layer. In comparison, our proposed method, ECG-Mamba, significantly outperforms these approaches, demonstrating superior performance in terms of AUPRC and AUROC on public datasets (PhysioNet / CinC Challenges of 2020 and 2021).

### B. Mamba Models

The Structured State Space Sequence (S4) [19] is a new architecture that offers a viable alternative to transformers and CNNs. It achieves state-of-the-art results in long-range sequence tasks by using an SSM. Mamba [10] is a Selective Scan Space State Sequential Model that builds upon S4 by incorporating a hardware-aware algorithm and an input-dependent selection mechanism. This allows for linear complexity without sacrificing global receptive fields, particularly in discrete data modelling. Vim [11] integrates SSMs with visual data processing, showcasing its adaptability. The proposed ECG-Mamba model adapts Vim for ECG signal processing, customising it for heart disease classification while addressing the challenges of ECG data analysis.

### C. Data Augmentation Methods

MixUp [15] and CutMix [16] are widely used techniques to improve the generalization of the model. MixUp is a data augentation method that creates new training samples by blending pairs of images and their labels using linear interpolation. In contrast, CutMix creates synthetic samples by cutting and pasting patches between training images and adjusting their labels proportionally to the area of the patches. Both methods aim to improve model performance and generalization by diversifying the training data. The proposed Non-Uniform-Mix strategy builds on these techniques by applying MixUp selectively to different parts of the dataset across training epochs, adopting a conservative augmentation approach.

## III. Algorithm Architecture

### A. Preliminaries

This section presents a Mamba-based approach for automated heart disease diagnosis using ECG data. The theoretical foundations of the Mamba architecture and Vim, which serve as the core components of the proposed diagnostic framework, are introduced here.

Mamba represents a paradigm shift in sequence modeling by introducing a SSM architecture that addresses the limitations of traditional attention-based transformers. While transformers compute attention across all token pairs with quadratic complexity $O(n^{2})$, Mamba processes sequences linearly $O(n)$ through selective state spaces. The Mamba model utilizes an SSM to capture temporal dynamics in ECG signals. The continuous-time SSM is defined as:

\begin{align*} \dot {x}(t) & = A(t)x(t)+Bu(t), \\ y(t) & = Cx(t)+Du(t), \tag {1}\end{align*}

where $x(t) \in \mathbb {R}^{N}$ represents the hidden state, $u(t) \in \mathbb {R}^{d}$ the input sequence, $y(t) \in \mathbb {R}^{d}$ the output, $A(t) \in \mathbb {R}^{N \times N}$ is an input-dependent state transition matrix, $B \in \mathbb {R}^{N \times d}$ is the input projection, $C \in \mathbb {R}^{d \times N}$ is the output projection, and $D \in \mathbb {R}^{d \times d}$ is set to zero $(D = 0)$.

To enable practical implementation in the Mamba architecture, this model is discretized using the zero-order hold (ZOH) method with a time step $\Delta t$. The resulting discrete-time SSM is:

\begin{align*} h[n+1] & = A_{d} h[n] + B_{d} u[n], \\ y[n] & = C_{d} h[n], \tag {2}\end{align*}

where $A_{d} = e^{A\Delta t}$, $B_{d} = \int _{0}^{\Delta t} e^{A\tau } B {\,}d\tau $, and $C_{d} = C$. In the implementation, the Mamba model uses a structured SSM with optimized parameters, as described in [10].

Vim extends the Mamba architecture to computer vision tasks by adapting the selective SSM for processing 2D visual data. The architecture treats image patches as sequential tokens and processes them using a hierarchical structure of Mamba blocks. Each block consists of:

• A patch embedding layer that converts image patches into sequential representations
• Selective state space layers that capture both local and global visual dependencies
• Multi-layer perceptions for feature transformation

The defining feature of Vim is its ability to model spatial relationships efficiently without the need for explicit attention mechanisms. The model achieves this through bidirectional sequential processing along both height and width dimensions of the feature maps, effectively capturing 2D spatial contexts. This approach has demonstrated competitive performance on various vision tasks while maintaining the linear computational complexity characteristic of the original Mamba architecture.

### B. ECG Sample Pre-Processing

All signals are resampled to 500 Hz. Polyphase filtering is applied to downsample 1000 Hz signals, effectively reducing the sampling rate while minimizing aliasing and preserving signal quality. For the 257 Hz signals, the Fast Fourier Transform (FFT) is utilized to upsample to 500 Hz, efficiently transforming the frequency domain representation to match the desired sampling rate. As stated in [13], the signals are zero-padded to a length of 8192 samples. Signals exceeding this length are randomly sampled and truncated to 8192 samples. Finally, each signal undergoes z-score standardization, as also utilized in [13], for normalization.

### C. Non-Uniform-Mix

In this research, a new data augmentation method is proposed for classifying heart abnormalities in 12-lead ECGs. The method, termed Non-Uniform-Mix, differs from existing techniques such as MixUp and CutMix by employing a gradual and non-uniform approach to mixing ECG samples and interpolating labels. Starting from the first epoch, 20% of the training data undergoes the MixUp operation. This proportion increases by 20% in each subsequent epoch, reaching 80% by the fifth epoch, where it then remains constant. The samples and labels of the data points are interpolated as follows:

\begin{align*} X_{g} & = \lambda X_{c} + (1-\lambda )X_{r}, \\ L_{g} & = \lambda L_{c} + (1-\lambda )L_{r}, \tag {3}\end{align*}

$X_{c}$ is a certain sample, $X_{r}$ is a random sample, $L_{c}$ is the label of certain sample, $L_{r}$ is the label of random sample, $X_{g}$ is the generated sample, $L_{g}$ is the label of generated sample. The variable $\lambda $ is a random number drawn from the Beta distribution, i.e., $\lambda \in Beta(\alpha , \beta $), where $\alpha =10$ and $\beta =10$ are fixed parameters determined through several experiments, and also $0\leq \lambda \leq 1$. The random sample $X_{r}$ is selected randomly from the entire training set based on the Beta distribution.

The ratio of dataset participation in interpolation during each epoch is presented in Table 1. Each sample participates according to a uniform distribution. The interpolated samples are used directly in place of the original samples for each training batch, just like MixUp and CutMix. This progressive mixing strategy enables the Mamba model to capture more detailed features while minimizing the impact of noise, thereby enhancing classification performance.

**TABLE 1:** The Ratio of Dataset Participation in Interpolation During Each Epoch

epoch	1	2	3	4, 5, 6, 7, …
ratio	20%	40%	60%	80%

### D. ECG-Mamba

**FIGURE 1.**
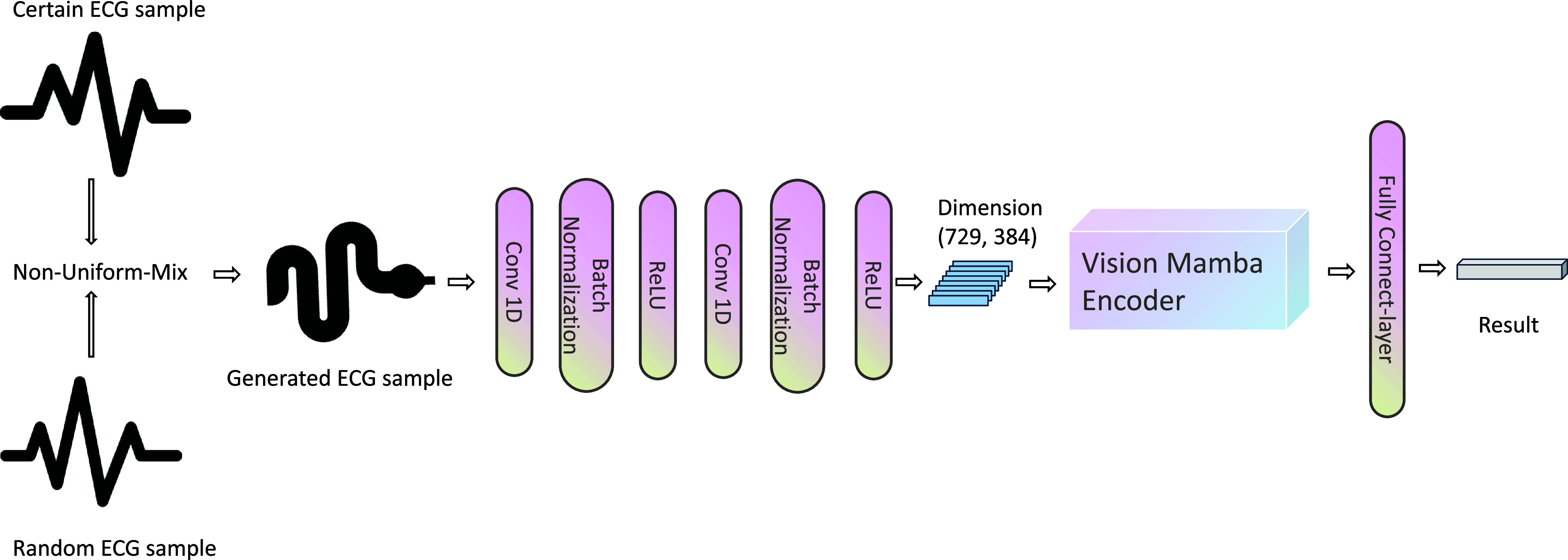
The ECG-mamba architecture and non-uniform-mix method.

Vim, originally designed to process 2-dimensional visual data, is adapted to handle multidimensional 12-lead ECG signals, which can be represented as a $12 \times N$ tensor, where 12 is the number of leads and *N* is the number of time samples. To facilitate this adaptation, two 1D convolutional neural network (CNN) layers are incorporated before the Vim encoder. These CNN layers extract features from the multichannel ECG signals, transforming them into a format compatible with the Vim encoder for further processing. After each CNN layer, batch normalization and the ReLU activation function are applied to improve learning efficiency and model performance. The architecture is illustrated in Figure 1, and the configuration of the two convolutional layers is detailed in Table 2. In [11], the best results were achieved by fine-tuning with an input size of (729, 384) and 24 Vim encoder blocks.

**TABLE 2:** The Configuration of Convolution Layers for the ECG Dataset

architecture	input size	output size	kernal size	stride	padding
Conv1d	12	128	14	3	2
BatchNorm1d	128	128			
ReLU	128	128			
Conv1d	128	384	15	4	100
BatchNorm1d	384	384			
ReLU	384	384

## IV. Experiments

### A. Dataset Description

For the experiments, datasets from the PhysioNet/CinC Challenges of 2020 and 2021 were used, comprising 88,253 individual records from seven institutions across four countries spanning three continents. These datasets, reflecting diverse demographics, disease severity levels, and comorbidities, enable rigorous clinical validation to ensure ECG-Mamba’s safety, effectiveness, and accuracy in real-world clinical settings.

The 2020 PhysioNet/CinC Challenge [20] focused on developing algorithms to automatically identify clinical diagnoses from 12-lead ECG recordings, emphasizing the accuracy and reliability of ECG classification—a critical component of diagnosing various heart conditions. The 2021 challenge [21] expanded this scope to include ECG recordings with different lead configurations, such as 12-lead, 6-lead, 4-lead, 3-lead, and 2-lead formats, aiming to develop algorithms capable of handling diverse ECG lead combinations. The 2021 dataset entirely contains the 2020 dataset, meaning that everything present in the 2020 dataset is also included in the 2021 dataset. This overlap is why the current study primarily uses the 2021 dataset, taking advantage of its broader scope. Both challenges aimed to advance automated ECG analysis by encouraging innovative and effective solutions for cardiac health monitoring.

The 2021 dataset includes 26 categories, comprising 25 cardiac abnormalities and 1 sinus rhythm, whereas the 2020 dataset includes 27 categories, comprising 26 cardiac abnormalities and 1 sinus rhythm. Additionally, the 2021 dataset contains approximately twice as much data as the 2020 dataset, with a total of 43,101 records.

For consistency in comparison, most simulations were performed using the 2021 dataset. However, the 2020 dataset was used to compare results with the best-performing method [12] from the 2020 challenge.

The 2021 dataset contains signals sampled at three frequencies: 500 Hz, 1000 Hz, and 257 Hz. It includes 87,663 samples at 500 Hz, 516 samples at 1000 Hz, and 74 samples at 257 Hz. A detailed configuration of the data used in the experiments is provided in Table 3.

**TABLE 3:** Overview of ECG Datasets Used in the Proposed Strategy: All Datasets Sourced from the Official PhysioNet/CinC Challenge 2021

Dataset Name	Number of Files	Each File Duration (sec)	Frequency (Hz)
CPSC and CPSC-Extra Database	10330	6-144	500
St Petersburg INCART 12-lead Arrhythmia Database	74	1800	257
PTB and PTB-XL databases	22353	10-120	500 or 1000
Georgia database	10344	5-10	500
Ningbo First Hospital	34905	10	500
Chapman University, Shaoxing People’s Hospital	10247	10	500
All Combined Datasets	88253	–	–

### B. Experimental Setup

For the experiments, the PyTorch library was utilized, and the training was conducted on a GeForce RTX 3090 Ti GPU with 24GB of memory. The GPU driver is based on version 535, with CUDA version 11.8. The operating system used is Ubuntu 22.04.

### C. Dataset Division: PhysioNet/CinC Challenge 2021

The data was divided into training and testing datasets using stratified proportional allocation and random shuffling. This approach was necessary to account for the 26 different classes and the unbalanced nature of the data. Four-fifths of the dataset was allocated to the training set, while the remaining one-fifth was used for testing. Instead of creating a separate validation set, a 5-fold cross-validation approach was adopted to efficiently and robustly evaluate the model’s performance across multiple subsets of the data. This method maximized the use of available data for training, particularly in the context of limited or unbalanced datasets. All experiments were conducted using this 5-fold cross-validation setup, with 70,602 records for training and 17,651 records for testing.

### D. Dataset Division: PhysioNet/CinC Challenge 2020

The training and test sets comprised 80% (34,481 records) and 20% (8,620 records) of the data, respectively, using stratified proportional allocation and random shuffling for a 5-fold cross-validation approach, which generated five outcomes that were averaged to produce a single result. This 5-fold cross-validation was employed to ensure robust model evaluation by reducing variance and providing a reliable estimate of performance across multiple data subsets.

### E. Learning Rate Schedule

To optimize the model, we used the Adam optimizer [22], which adjusts the learning rate individually for each parameter. Additionally, we implemented the Noam schedule [23], providing a global learning rate that varies dynamically based on the training step. The training process included a warm-up phase of 4,000 steps, during which the learning rate was gradually increased. Following the warm-up phase, the learning rate decreased progressively. With a batch size of 30, this setup resulted in 2,353 steps per epoch during training.

The learning rate $lr(t)$ at step *t* is defined as:

\begin{equation*} lr(t) = d_{model}^{-0.5} \cdot \min (t^{-0.5},~t \cdot \omega ^{-1.5}), \tag {4}\end{equation*}

$d_{model}$ is the dimensionality of the model embedding and is set to 729. Although the embedding size for ECG-Mamba is 384, a sequence length of 729 was found to yield better performance. The variable *t* represents the current training step, while $\omega $ is a hyperparameter that controls the duration of the warm-up phase, during which the learning rate gradually increases before transitioning to a decay phase.

### F. Evaluation Metric

The performance of the ECG classification model is primarily evaluated using three metrics to address the imbalanced and multi-label nature of the dataset.

• *Macro Area Under the Precision-Recall Curve (AUPRC):* This metric provides a comprehensive measure of performance across all classes, with a particular focus on the model’s ability to handle minority classes effectively. AUPRC is well-suited for datasets with significant class imbalances, as it emphasizes the precision and recall of underrepresented classes.
• *Macro Area Under the Receiver Operating Characteristic Curve (AUROC):* AUROC evaluates the classifier’s ability to distinguish between positive and negative instances, giving equal weight to each class. This metric ensures the model’s overall discrimination capability is assessed regardless of class proportions.
• *Challenge Score:* Developed as part of the official PhysioNet/CinC Challenges of 2020 and 2021, the Challenge Score is a specialized metric designed for ECG classification. It goes beyond traditional accuracy by incorporating clinical relevance, assigning partial credit for predictions of clinically similar diagnoses. Clinically dangerous misclassifications are penalized more heavily, while errors between similar conditions are treated more leniently. This approach ensures the evaluation aligns with expert cardiologists’ perspectives and prioritizes patient safety and treatment outcomes.

These metrics collectively ensure a robust evaluation of the model’s performance across the varied and imbalanced dataset. AUPRC and AUROC are particularly valuable for assessing the impact of data augmentation methods on model robustness and class balance. Meanwhile, the Challenge Score provides a clinically meaningful perspective, crucial for comparing against state-of-the-art algorithms from the PhysioNet/CinC Challenges.

When comparing the proposed model with published results from the PhysioNet/CinC Challenges of 2020 and 2021, all three metrics (AUPRC, AUROC, and Challenge Score) are employed. This comprehensive approach ensures the evaluation captures both technical and clinical aspects of performance, providing a well-rounded assessment of the model’s effectiveness.

## V. Results and Analysis

To analyze the performance of ECG-Mamba, its behavior was evaluated under different learning rate schedules: Step Learning Rate Scheduler (StepLR) and Noam. In the best method from the PhysioNet/CinC Challenge 2021 [13], StepLR was employed to globally update the learning rate, starting with an initial rate of 0.001 and decaying by a factor of 0.1 every 20 epochs. When this StepLR schedule is applied to ECG-Mamba, as shown in Table 4, the model achieved an AUPRC of 0.5158 and an AUROC of 0.9403.

**TABLE 4:** Performance Analysis of ECG-mamba Using Noam Scheduler (with Warm-up Steps) Versus StepLR Scheduler

Learning Rate Schedule	Warm-up step	AUPRC	AUROC
StepLR	0	0.5158	0.9403
Noam	8000	0.6025	0.9633
Noam	4000	0.6100	0.9643
Noam	2000	0.6092	0.9634
Noam	1000	0.5926	0.9607

**FIGURE 2.**
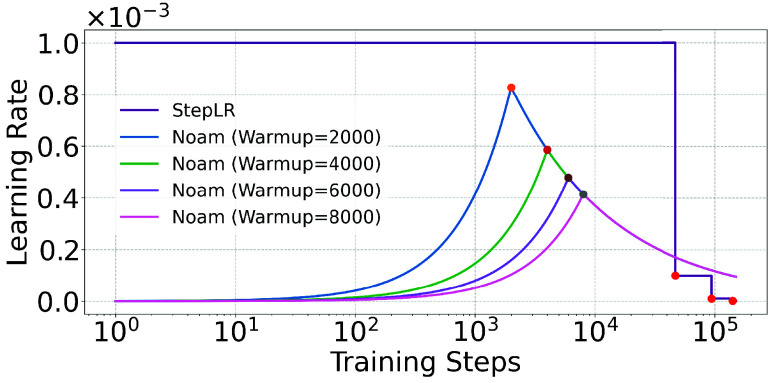
Learning rate dynamics: Noam scheduler (different warm-up steps) vs StepLR over training steps.

When Noam is used as the learning rate schedule, as seen in Table 4, the AUPRC increases by approximately 20%, and the AUROC improves by about 2% compared to StepLR. Through several experiments, we determined that setting the warm-up step to 4,000 yields optimal AUPRC and AUROC values of 0.6100 and 0.9643, respectively. These results presented in Table 4 highlight the significant impact of the learning rate schedule on the performance of ECG-Mamba. Furthermore, the learning rate variations over training steps for both Noam and StepLR schedules are depicted in Figure 2, illustrating how the warm-up phase in the Noam schedule contributes substantially to improved model performance.

To demonstrate the potential of ECG-Mamba in medical diagnostics for 12-lead ECG classification, comparisons are made with the best methods from the PhysioNet/CinC Challenge 2020 and 2021, including ResNet without attention. The best method’s hyperparameters for the PhysioNet/CinC Challenge 2021 align with [13], and those for 2020 align with [12].

The PhysioNet/CinC Challenge 2020 featured a dataset comprising 12-lead ECG recordings. The top method, developed by team Prna [12], utilised a Transformer architecture and achieved outstanding results with an AUPRC of 0.5115, an AUROC of 0.9318, and a challenge score of 0.5798, as shown in Table 5. However, ECG-Mamba surpassed these results by achieving an AUPRC of 0.5452 and an AUROC of 0.9497, with a significantly higher challenge score of 0.7270, consequently outperforming the previous best method across all evaluation metrics.

**TABLE 5:** Comparing ECG-Mamba with the Best Methods from PhysioNet/CinC Challenge 2020 (by Team Prna) and Challenge 2021 (by Team ISIBrno), and the Original Mamba

Dataset	Method	Loss Function	# of Parameters	AUPRC	AUROC	Challenge Score
Challenge 2020	Prna	Binary Cross Entropy	13.64 million	0.5115	0.9318	0.5798
	ECG-Mamba (24 blocks)	Binary Cross Entropy	26.09 million	0.5452	0.9497	0.7270
Challenge 2021	ISIBrno (Random-lead)	Mixture	6.56 million	0.5041	0.9036	0.7116
	ISIBrno	Mixture	6.56 million	0.5230	0.8960	0.7165
	ISIBrno w/o attention	Mixture	6.29 million	0.5452	0.8863	0.6967
	mamba (24 blocks)	Binary Cross Entropy	24.19 million	0.5348	0.9455	0.6350
	ECG-Mamba (5 blocks)	Binary Cross Entropy	6.26 million	0.5890	0.9601	0.6941
	ECG-Mamba (24 blocks)	Binary Cross Entropy	26.09 million	0.6100	0.9643	0.7013
	ECG-Mamba (24 blocks) w/o normalization	Binary Cross Entropy	26.09 million	0.6196	0.9670	0.7130
	ECG-Mamba (24 blocks) + Non-Uniform-Mix	Binary Cross Entropy	26.09 million	0.6271	0.9671	0.7195

In the PhysioNet/CinC Challenge 2021, the training dataset included random lead configurations (e.g., 12-lead, 6-lead, 4-lead, 3-lead, and 2-lead). The best-performing method, developed by team ISIBrno [13], based on the Residual Network (ResNet), achieved an AUPRC of 0.5040 and an AUROC of 0.9036. Since 12-lead ECG is the focus of this study, the best method was evaluated using 12 leads, as shown in Table 5. This resulted in an approximately 3.7% increase in AUPRC but a 0.84% decrease in AUROC. Nejedly et al. further modified the ResNet by removing the attention layer (ISIBrno without attention) [14], leading to a 4.2% increase in AUPRC to 0.5452, but a 1.3% decrease in AUROC to 0.8863. The ResNet achieved a challenge score of 0.7116, which improved slightly to 0.7165 with 12-lead data. This higher challenge score is attributed to the mixture loss strategy, which consists of three components: binary cross-entropy, a custom challenge loss [24], and a custom sparsity loss [13]. The custom challenge loss serves as a differentiable approximation of the challenge score, encouraging the network to maximize it while considering class weights. The sparsity loss, based on a parabolic function, penalizes the network for generating probability values near 0.5, promoting outputs that are closer to 0 or 1. This helps improve the optimization of the final decision threshold.

The ECG-Mamba achieved a challenge score of 0.7013, slightly lower than the best result from ResNet. However, applying the Non-Uniform-Mix data augmentation method to ECG-Mamba significantly increased the challenge score to 0.7195, outperforming the ResNet, including its mixture loss strategy. Additionally, the proposed ECG-Mamba method outperformed these methods in terms of AUPRC and AUROC, achieving an AUPRC of 0.6100 and an AUROC of 0.9643. ECG-Mamba increased the AUPRC by 11.8% and the AUROC by 8.8% compared to the ResNet without attention.

The ECG-Mamba shows a significant improvement over the original Mamba. The primary distinction lies in the inclusion of bidirectional blocks in ECG-Mamba, which handle both forward and backward dependencies in visual sequences. This upgrade enables the ECG-Mamba to capture global context and positional information more effectively. As shown in Table 5, the ECG-Mamba with bidirectional blocks achieves a 14% improvement in AUPRC and a 1.9% improvement in AUROC compared to the original Mamba, highlighting the effectiveness of the bidirectional architecture.

A comparison between ECG-Mamba and the best-performing algorithm from the PhysioNet/CinC Challenge 2021 (ISIBrno without attention [14]) was conducted using AUPRC and AUROC metrics.

As shown in Table 5, reducing the number of blocks in ECG-Mamba from 24 to 5 resulted in a decrease in AUPRC from 0.6100 to 0.5890 and AUROC from 0.9671 to 0.9601. However, this reduction also decreased the number of parameters to approximately one-fourth of the 24-block variant. Despite using fewer parameters than ResNet without attention, the 5-block ECG-Mamba still achieved better AUPRC and AUROC scores, further demonstrating the effectiveness of ECG-Mamba.

In this study, enhanced performance was observed across key metrics (AUPRC increased from 0.6100 to 0.6196, AUROC increased from 0.9643 to 0.9670, and challenge score increased from 0.7013 to 0.7130, as shown in Table 5) when z-score normalization was omitted, contrary to the standard practice in ECG classification literature, such as Prna and ISIBrno, where input data are typically standardized. This unexpected outcome suggests that the preserved amplitude of the ECG signal, which normalization alters, contains diagnostic information critical for classification. However, for a side-by-side comparison with PRNA and ISIBrno, z-score normalization was applied in an additional experiment.

To demonstrate the effectiveness of the proposed Non-Uniform-Mix strategy, it is compared with CutMix and MixUp. CutMix is employed in two different ways: one with a fixed truncation length $(\alpha)$ set as a percentage of the total length, following [25] where $\alpha = 0.2$; the other with a random $\alpha $. As indicated in Table 6, both AUPRC and AUROC decrease significantly when the $\alpha $ value is random, with AUPRC dropping to 0.5761 and AUROC to 0.9539 compared to the baseline. When the $\alpha = 0.2$, AUPRC decreases to 0.6093, and AUROC decreases to 0.9630. Additionally, when MixUp is used as the data augmentation method, both AUPRC and AUROC drop to 0.6042 and 0.9616, respectively, compared to the baseline. While both MixUp and CutMix( $\alpha = 0.2$) lead to decreased performance, the decline in AUPRC and AUROC is less severe with CutMix ( $\alpha = 0.2$). These results suggest that ECG-Mamba may be sensitive to noise, as data augmentation methods typically introduce noise into the model [26].

**TABLE 6:** Comparison of Different Configurations of Non-Uniform-Mix and other Data Augmentation Methods Applied to ECG-mamba on the PhysioNet/CinC Challenge 2021 Dataset

Data Augmentation Method	AUPRC	AUROC
Baseline	0.6100	0.9643
CutMix ( $\alpha = random$)	$\downarrow \downarrow 0.5761$	$\downarrow \downarrow 0.9539$
CutMix ( $\alpha = 0.2$)	$\downarrow ~0.6093$	$\downarrow ~0.9630$
MixUp	$\downarrow ~0.6042$	$\downarrow ~0.9616$
Non-Uniform-Mix (cyclicality)	$\uparrow ~0.6186$	$\uparrow ~0.9660$
Non-Uniform-Mix (Start: 1st epoch $\rightarrow ~100$%)	$\downarrow ~0.6051$	$\downarrow ~0.9622$
Non-Uniform-CutMix (Start: 1st epoch $\rightarrow ~80$%)	$\uparrow ~0.6183$	$\uparrow ~0.9652$
Non-Uniform-Mix (Start: 6th epoch, 80%)	$\uparrow ~0.6209$	$\uparrow ~0.9669$
Non-Uniform-Mix (Start: 1st epoch $\rightarrow ~80$%)	$\uparrow \uparrow $ 0.6271	$\uparrow $ 0.9671

Legend: $\uparrow$ indicates that the result has improved compared to the baseline, while $\downarrow$ indicates a decline in performance. A substantial decline is indicated by $\downarrow \downarrow$. Similarly, $\uparrow \uparrow$ indicates a substantial improvement in performance.

The core idea of Non-Uniform-Mix is to determine the optimal range for applying data augmentation methods at each epoch. Unlike widely used methods such as MixUp and CutMix, which interpolate data uniformly, Non-Uniform-Mix partially mixes the data and progressively increases the augmentation range throughout training. A cycling strategy is also implemented, where the augmentation range for each epoch follows patterns such as (0%, 20%, 40%, 0%), (0%, 60%, 80%, 0%), and (80%, 80%, 80%, 80%, …). The ellipsis (...) represents the rest of the epochs during training, where the data augmentation method, Mix-Up, is applied to 80% of the training dataset. In this cycling strategy, inspired by [27], the dataset participation in interpolation gradually increases overall, though some epochs do not employ data augmentation. As shown in Table 6, this cycling approach leads to slight increases in both AUPRC and AUROC.

For Non-Uniform-Mix with progressively increasing augmentation range, Table 6 shows that when MixUp is applied at 80% after three epochs, AUPRC significantly improves by 2.8% (reaching 0.6271) compared to the baseline, while AUROC shows a slight increase. However, when the augmentation range is progressively increased to 100% until the end of training, both AUPRC and AUROC decline to 0.6051 and 0.9622, respectively.

An alternative configuration of Non-Uniform-Mix was tested to explore the effect of reduced participation in the training phase. When MixUp was applied only after five epochs, as seen in Table 6, both AUPRC and AUROC showed slight improvements over the baseline.

For a broader comparison, CutMix was also applied progressively and uniformly, labelled as Non-Uniform-CutMix in Table 6. The results show improvements in both AUPRC and AUROC, indicating that progressive application of augmentation methods can enhance ECG data analysis. This demonstrates the effectiveness of Non-Uniform-Mix, though it should be noted that setting 100% participation during training can lead to decreased performance in ECG-Mamba due to excessive noise.

**FIGURE 3.**
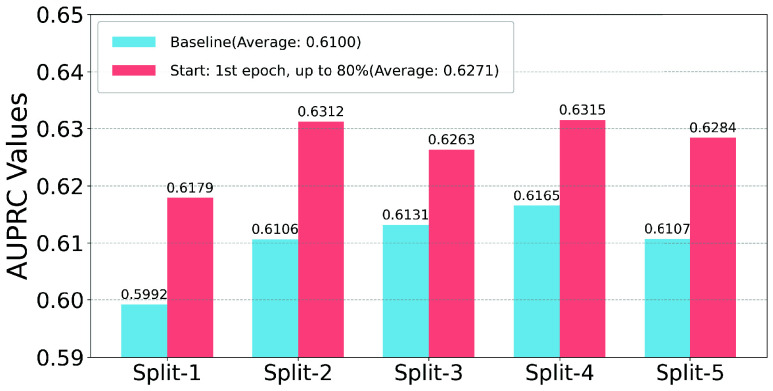
Comparison of the best non-uniform-mix configuration with baseline AUPRC values across 5-Fold cross-validation splits.

**FIGURE 4.**
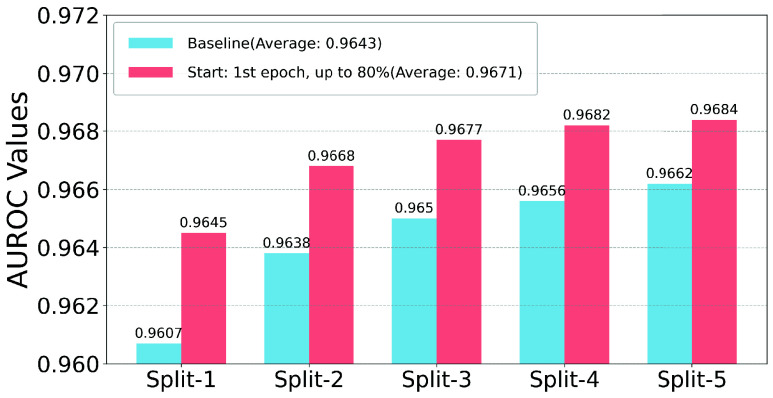
Comparison of the best non-uniform-mix configuration with baseline AUROC values across 5-fold Cross-Validation Splits.

To demonstrate the overall performance of Non-Uniform-Mix for AUPRC and AUROC, Figures 3 and 4 compare the best Non-Uniform-Mix configuration with baseline AUPRC and AUROC values across 5-fold cross-validation splits. As shown in the figures, Non-Uniform-Mix significantly improves the ECG-Mamba’s performance on AUPRC across five different dataset configurations. Although the baseline AUROC values are already very high, Non-Uniform-Mix further enhances them.

Non-Uniform-Mix was also applied to other architectures. As shown in Table 7, the Transformer, which is the architecture of the best method Prna on the PhysioNet/CinC Challenge 2020 Dataset, was deployed with three data augmentation methods. However, its performance decreased in terms of AUPRC and AUROC on the PhysioNet/CinC Challenge 2020 dataset when MixUp and CutMix ( $\alpha =0.2$) augmentations were used, suggesting that the Prna is more sensitive to noise, as these data augmentation methods may introduce additional noise that adversely impacts its performance. In contrast, the performance increased slightly with the proposed Non-Uniform-Mix method, which is based on MixUp, achieving an AUPRC of 0.5129 and an AUROC of 0.9334. The non-uniform operations in Non-Uniform-Mix appear to mitigate the introduction of noise, leading to improved performance compared to standard MixUp.

**TABLE 7:** Comparison of Non-Uniform-Mix with other Data Augmentation Methods Applied to the Best Method on the PhysioNet/CinC Challenge 2020 Dataset

Method	Data Augmentation Method	AUPRC	AUROC
Prna	None	0.5115	0.9318
	MixUp	0.4908	0.9280
	CutMix( $\alpha = 0.2$)	0.4642	0.9169
	Non-Uniform-Mix	0.5129	0.9334

In Table 8, a similar trend is observed for the ResNet architecture, which is the architecture of the best method ISIBrno on the PhysioNet/CinC Challenge 2021 Dataset. However, for CutMix ( $\alpha = 0.2$), the AUROC improved for ISIBrno without attention, indicating that CutMix ( $\alpha = 0.2$) helps the model better distinguish between classes overall. Despite this, the precision decreased (reflected by a lower AUPRC), likely due to the introduction of more false positives.

**TABLE 8:** Comparison of Non-Uniform-Mix with other Data Augmentation Methods Applied to Original Mamba and ISIBrno (Without Attention) on the PhysioNet/CinC Challenge 2021 Dataset

Method	Data Augmentation Method	AUPRC	AUROC
ISIBrno w/o attention	None	0.5452	0.8863
	MixUp	0.4547	0.8815
	CutMix( $\alpha = 0.2$)	0.4966	0.9141
	Non-Uniform-Mix	0.4775	0.8854
mamba (24 blocks)	None	0.5348	0.9455
	MixUp	0.5242	0.9380
	CutMix( $\alpha = 0.2$)	0.5343	0.9410
	Non-Uniform-Mix	0.5452	0.9456

The comparison of Non-Uniform-Mix with other data augmentation methods applied to the original Mamba on the PhysioNet/CinC Challenge 2021 dataset is presented in Table 8. Both AUPRC and AUROC decreased when MixUp and CutMix were used. However, Non-Uniform-Mix improved the Mamba’s performance in both AUPRC and AUROC. This improvement is not limited to ECG-Mamba; the original Mamba also benefits from this data augmentation method. These results suggest that Non-Uniform-Mix is well-suited for the Mamba architecture when working with ECG data. In contrast, Non-Uniform-Mix showed a slight performance increase on the Transformer architecture but did not perform well on the ResNet architecture, indicating limitations in its applicability across different model architectures. This observation suggests a direction for future research, specifically in developing tailored data augmentation methods optimized for ResNet architectures and further enhancing performance on Transformer architectures to effectively handle complex ECG datasets, such as those from the PhysioNet/CinC Challenges (2020 and 2021).

## VI. Conclusion

The proposed ECG-Mamba model, based on the Vim architecture, demonstrates significant potential in detecting heart abnormalities from ECG. Utilising the bidirectional SSM of Mamba, ECG-Mamba effectively processes the sequential nature of ECG data, outperforming leading algorithms from the PhysioNet/CinC Challenges of 2020 and 2021. However, the model’s sensitivity to noise highlights the need for careful data augmentation strategies. The introduction of a conservative data augmentation algorithm, which applies MixUp selectively across different epochs, has further improved ECG-Mamba’s performance. These results indicate that ECG-Mamba, with its tailored augmentation approach, represents a promising advancement in the application of SSMs for ECG-based diagnostics, offering both improved accuracy and robustness.

## VII. Clinical Impact

Beyond technical advancements, ECG-Mamba shows substantial clinical impact. Through retrospective validation using clinical data, the model has demonstrated its potential to support clinical decision-making by providing cardiologists with precise detection of heart abnormalities, enabling earlier interventions. This improved diagnostic precision contributes to better patient outcomes, particularly for conditions requiring urgent attention, such as arrhythmias. Additionally, ECG-Mamba’s efficient processing of ECG data streamlines workflow in busy clinical environments, reducing the time required for manual ECG interpretation and optimising resource utilisation. By integrating seamlessly into existing diagnostic pipelines, the model enhances the overall quality of care, offering a scalable solution for hospitals and clinics. These results suggest that ECG-Mamba, with its tailored augmentation approach and proven clinical utility, represents a transformative advancement in the application of SSMs for ECG-based diagnostics.

## VIII. Robustness and Generalization

ECG-Mamba demonstrates robustness and generalisation, critical for reliable performance in diverse clinical scenarios. The model was trained and validated on the PhysioNet/CinC Challenges datasets, which include ECG signals with varying sampling rates, lead configurations, and clinical conditions, ensuring resilience to data quality variations and noise. The Non-Uniform-Mix augmentation strategy further enhances robustness by introducing controlled variability during training, enabling the model to handle challenging inputs effectively. Rigorous testing through 5-fold cross-validation across diverse demographics and disease severities confirms ECG-Mamba’s ability to generalise to unseen data, supporting its applicability in real-world clinical settings with complex and heterogeneous patient populations.

## IX. Ethical and Regulatory Compliance

ECG-Mamba meets ethical and regulatory requirements for healthcare AI systems. The model was developed using anonymised datasets from the PhysioNet/CinC Challenges, ensuring no personal or health-related information is processed, in line with the Health Insurance Portability and Accountability Act (HIPAA) in the United States and the General Data Protection Regulation (GDPR) in Europe. To ensure fairness, ECG-Mamba leverages diverse datasets covering varied demographics and health conditions across multiple institutions and countries, promoting equitable performance across patient groups. For future clinical use, processes such as obtaining patient consent and assessing data processing risks will be implemented to comply with healthcare regulations. These efforts ensure ECG-Mamba’s safe and responsible integration into clinical practice, supporting high standards of patient care and trust.
